# Photocatalysts Based on Graphite-like Carbon Nitride with a Low Content of Rhodium and Palladium for Hydrogen Production under Visible Light

**DOI:** 10.3390/nano13152176

**Published:** 2023-07-26

**Authors:** Angelina V. Zhurenok, Danila B. Vasichenko, Semen N. Berdyugin, Evgeny Yu. Gerasimov, Andrey A. Saraev, Svetlana V. Cherepanova, Ekaterina A. Kozlova

**Affiliations:** 1 Federal Research Center, Boreskov Institute of Catalysis SB RAS, Lavrentieva Ave. 5, Novosibirsk 630090, Russia; angelinazhurenok@gmail.com (A.V.Z.); vasilchenko@niic.nsc.ru (D.B.V.); gerasimov@catalysis.ru (E.Y.G.); asaraev@catalysis.ru (A.A.S.); svch@catalysis.ru (S.V.C.); 2 Nikolaev Institute of Inorganic Chemistry, Siberian Branch of the Russian Academy of Science, Novosibirsk 630090, Russia; berdyugin@niic.nsc.ru

**Keywords:** photocatalysis, hydrogen evolution, g-C_3_N_4_, noble metals, rhodium, palladium

## Abstract

In this study, we proposed photocatalysts based on graphite-like carbon nitride with a low content (0.01–0.5 wt.%) of noble metals (Pd, Rh) for hydrogen evolution under visible light irradiation. As precursors of rhodium and palladium, labile aqua and nitrato complexes [Rh_2_(H_2_O)_8_(μ-OH)_2_](NO_3_)_4_∙4H_2_O and (Et_4_N)_2_[Pd(NO_3_)_4_], respectively, were proposed. To obtain metallic particles, reduction was carried out in H_2_ at 400 °C. The synthesized photocatalysts were studied using X-ray diffraction, X-ray photoelectron spectroscopy, UV–Vis diffuse reflectance spectroscopy and high-resolution transmission electron microscopy. The activity of the photocatalysts was tested in the hydrogen evolution from aqueous and aqueous alkaline solutions of TEOA under visible light with a wavelength of 428 nm. It was shown that the activity for the 0.01–0.5% Rh/g-C_3_N_4_ series is higher than in the case of the 0.01–0.5% Pd/g-C_3_N_4_ photocatalysts. The 0.5% Rh/g-C_3_N_4_ sample showed the highest activity per gram of catalyst, equal to 3.9 mmol g_cat_^–1^ h^–1^, whereas the most efficient use of the metal particles was found over the 0.1% Rh/g-C_3_N_4_ photocatalyst, with the activity of 2.4 mol per gram of Rh per hour. The data obtained are of interest and can serve for further research in the field of photocatalytic hydrogen evolution using noble metals as cocatalysts.

## 1. Introduction

Due to the growth of economic and environmental problems associated with the use of fossil fuels [1,2], the demand for a transition to alternative energy is growing [3]. Particular attention should be paid to hydrogen as a replacement for traditional energy sources [4]. Hydrogen is an environmentally friendly fuel and does not emit toxic compounds during combustion [5]. One of the most well-known processes for producing hydrogen used in industry is steam reforming of natural gas [6]. Despite the efficiency of hydrogen production of this method, steam reforming is not an environmentally friendly process, since it is energy-consuming, carried out at high temperatures and pressures and does not exclude CO_2_ emissions [7]. In this regard, photocatalytic hydrogen production is of particular interest [8,9]. In the 1970s, photocatalytic hydrogen production was first discovered by Fujishima and Honda by water splitting on semiconductor photocatalysts under light irradiation [10]. Solar energy is universal, and its reserves are unlimited [11], while photocatalytic hydrogen production is a process occurring under environmental conditions [12,13,14]. Thus, photocatalytic hydrogen production can be an effective solution for converting solar energy into hydrogen energy [8].

The main factor hindering the practical use of photocatalytic processes is the lack of efficient and at the same time stable heterogeneous photocatalysts operating under the action of visible light, which makes up about 43% of the solar spectrum [10]. Recently, the graphitic carbon nitride g-C_3_N_4_ has attracted more attention from researchers [15]. This material has the properties of a semiconductor with a band gap of 2.7 eV and positions of the valence and conduction bands suitable for the photocatalytic water splitting [16]. One of the common methods for increasing the activity of g-C_3_N_4_, as well as other semiconductor photocatalysts, is the deposition of metal co-catalysts on the surface of semiconductors [17,18], which leads to the spatial separation of electron–hole pairs. Traditionally, different metals, including noble metals, like Fe, Mn, Zn, Ni, Ag, Au, Cu and Pt, are used as co-catalysts [19,20].

In general, all platinum group metals can act as cocatalysts for photocatalytic hydrogen evolution [3]. However, for g-C_3_N_4_, among noble metals, platinum is usually deposited to the surface as a cocatalyst [21]. It is believed that platinum is the most effective co-catalyst for the formation of hydrogen; this metal forms the highest Schottky barrier at the metal–semiconductor interface, as it has the highest work function relative to other metals [8]. At the same time, it would be interesting to compare the deposition of other precious metals from the platinum group (especially Rh and Pd), since both experimental and theoretical results [22,23,24,25] presented for the HER suggest that these noble metals are located at the top of the corresponding volcano plot, and, therefore, according to the Sabatier principle they should provide near-optimal activity for hydrogen production. Also, in view of the high volatility of noble metals prices, it seems pragmatic to have information on their substitutability in catalysis. The literature describes only a few works on the photocatalytic production of hydrogen over Rh/g-C_3_N_4_ [26] and Pd/g-C_3_N_4_ [27], respectively. However, Rh- and Pd-supported g-C_3_N_4_-based catalysts have previously been shown to perform well in ammonia borane hydrolysis [28,29,30] and various photocatalytic oxidation processes [31,32,33].

In our previous research on the synthesis of platinized TiO_2_ and g-C_3_N_4_ for hydrogen production, the deposition of platinum by means of sorption of (Bu_4_N)_2_[Pt(NO_3_)_6_] labile complex followed by reduction in a stream of hydrogen allows one to obtain very active Pt/TiO_2_ and Pt/g-C_3_N_4_ photocatalysts. This approach has been proven to obtain highly dispersed states of noble metals (<1 nm), which are characterized by strong interaction with the support, which makes it possible to achieve high specific photocatalytic activity in target processes at a low metal content (up to 0.1 wt%) [34,35,36]. In this research, we aimed at the synthesis and investigation of photocatalysts based on graphitic carbon nitride with a low (0.01–0.5 wt%) content of noble metals (Pd, Rh) for hydrogen production under visible light. By analogy with platinized photocatalysts, highly labile complexes [Rh_2_(H_2_O)_8_(μ-OH)_2_](NO_3_)_4_∙4H_2_O and (Et_4_N)_2_[Pd(NO_3_)_4_] were used as precursors of rhodium and palladium, respectively; reduction in H_2_ at 400 °C was used to obtain metallic particles. The photocatalysts were tested in the photocatalytic production of hydrogen from aqueous and alkaline solutions of triethanolamine. Regularities were found for the activity and stability of Rh/g-C_3_N_4_ and Pd/g-C_3_N_4_ photocatalysts depending on the mass fraction of metals and the basicity of the medium.

## 2. Materials and Methods

### 2.1. Reagents

The following reagents were used: melamine (Aldrich, 98%, Burlington, MA, USA), cyanuric acid (Aldrich, 98%, USA), acetone (Ekos-1, 98%, Staraya Kupavna, Russia), RhCl_3_ (Krastsvetmet, 38.67% of Rh, Krasnoyarsk, Russia), pure Pd powder acid (Krastsvetmet, 99.99%, Krasnoyarsk, Russia).

### 2.2. g-C_3_N_4_ Synthesis

The proposed method for the synthesis of g-C_3_N_4_ consists of the thermolysis of the supramolecular melamine–cyanuric acid adduct [35]. Melamine (40.5 g, Aldrich, 98%) and cyanuric acid (41.5 g, Aldrich, 98%) were suspended in distilled water (300 mL) and heated at 90 °C for 12 h under continuous stirring. The suspension then was cooled, and a white solid was filtered off, which was then washed and dried in vacuum. The resulting material was placed in a muffle and kept at the temperature 550 °C for 1 h; the heating rate was 1 °C/min. The resulting light-yellow sample of g-C_3_N_4_ was ground in a mortar.

### 2.3. Rh and Pd Deposition

[Rh_2_(H_2_O)_8_(μ-OH)_2_](NO_3_)_4_∙4H_2_O (prepared from K_3_RhCl_6_ [37]) was dissolved in acetone + ethanol (3%) to obtain a solution with [Rh] of 0.568 mM (Rh solution). Also, (Et_4_N)_2_[Pd(NO_3_)_4_] (prepared from metallic palladium [38]) was dissolved in acetone to obtain a solution with [Pd] of 2.76 mM (Pd solution). An appropriate aliquot of Rh solution (11 mL for 0.5%, 2.2 mL for 0.1%, 1.1 mL for 0.05%, 0.22 mL for 0.01%), or Pd solution (4.8 mL for 0.5%, 0.96 mL for 0.1%, 0.48 mL for 0.05%, 0.096 mL for 0.01%), was added into a suspension of g-C_3_N_4_ (250 mg) in 1.5 mL of acetone. The resulting suspensions were thoroughly mixed for one (Pd) or five (Rh) days at room temperature until the mother liquor become colorless. The powder was separated by filtration, washed with acetone and dried in an air flow. The dried powders were heated in a stream of hydrogen at 400 °C for 1 h yielding the X%M/g-C_3_N_4_ (M = Rh or Pd) catalysts, where X = 0.5, 0.1, 0.05 or 0.01 and designates the percentage of corresponding metal (weight percent). For comparison, the deposition of 0.5% of Pd and Rh from solutions of [Pd(NH_3_)_4_(NO_3_)_2_] and RhCl_3_ was carried out using the same technique.

### 2.4. Photocatalyst Characterization

The photocatalysts were characterized by X-ray diffraction (XRD), X-ray photoelectron spectroscopy (XPS), UV–Vis spectroscopy and high-resolution transmission electron microscopy (HRTEM) techniques. XRD investigations was performed with a D8 Advance powder diffractometer (Bruker, Germany) using CuKα radiation. XRD patterns were recorded in the 2θ range from 20° to 80°. The mean sizes of crystallites were estimated from the full width at half maximum of the corresponding peaks using the Scherrer formula as follows:

(1) $$d=\frac{K\times \lambda }{\beta cos\theta }$$

where *d*—average size of coherent scattering crystallites (nm), *K*—Scherrer constant, *λ*—X-ray wavelength (nm), *β*—width of reflection at half height (2θ units), *θ*—diffraction angle.

XPS studies were carried out on a SPECS Surface Nano Analysis GmbH photoelectron spectrometer (Berlin, Germany) with monochromatic AlKα radiation. Diffuse reflectance spectra were recorded on a UV-2501 PC spectrophotometer (Shimadzu, Japan) equipped with an ISR-240A diffuse reflectance cell. The structure of the photocatalysts was studied by HRTEM using a ThemisZ electron microscope (Thermo Fisher Scientific, Waltham, MA, USA) operated at an accelerating voltage of 200 kV.

Elemental analysis was carried out for several samples with the ICP-AES technique with the use of an iCAP-6500 high-resolution spectrometer (Thermo Scientific, Waltham, MA, USA, (ICP-AES)) with a cyclone-type spray chamber and a SeaSpray nebulizer. The actual content of metals was in good agreement with calculated values (Table S1).

### 2.5. Photocatalytic Activity

The activity of the photocatalyst was investigated in a batch reactor. The reaction suspension containing 50 mg of a photocatalyst and 100 mL of an aqueous or aqueous alkaline (C_0_(NaOH (Aldrich, 98%)) 10 vol.% triethanolamine (TEOA, Reachim, 98%) solution was purged with argon for 30 min to remove oxygen before the start of the photocatalytic reaction. Then, the suspension was irradiated using an LED radiation source (*λ* = 428 nm, 56 mW/cm^2^). The area of the quartz window of the reactor was 22.5 cm^2^. The amount of hydrogen evolved was measured using a gas chromatograph (Khromos, Russia); the reaction duration was 90 min.

The apparent quantum efficiency (*AQE*) was calculated as follows:

(2) $$AQE=\frac{2\times W_{0}}{N_{ph}}\times 100\%$$

where *W*_0_—reaction rate (µmol min^−1^), *N_ph_*—photon flux constituting 283 µEinstein min^−1^. The number of electrons (2) involved in the hydrogen formation reaction was taken into account in the apparent quantum efficiency calculation.

## 3. Results

### 3.1. Photocatalyst Characterization

#### 3.1.1. X-ray Diffraction

**Figure 1:**
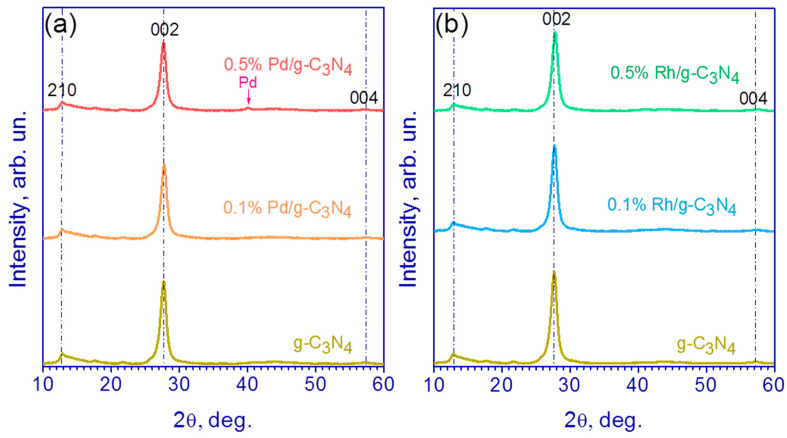
XRD patterns of pristine g-C_3_N_4_, (**a**) Pd (0.1 and 0.5 wt%)/g-C_3_N_4_ and (**b**) Rh (0.1 and 0.5 wt%)/g-C_3_N_4_.

In order to characterize the crystalline phase of the Pd/g-C_3_N_4_ and Rh/g-C_3_N_4_ samples, XRD patterns of the synthesized photocatalysts were obtained (Figure 1). Figure 1 shows that there are two main peaks in the XRD patterns, located at 13 and 27°; these peaks can be attributed to g-C_3_N_4_ [16]. The peak located at 13° corresponds to the reflection from the (210) plane and is due to the distance between the heptazine (tri-s-triazine) units in the 2D g-C_3_N_4_ layer, and the diffraction peak (2θ~27°) belongs to the (002) plane and determines the distance between the 2D layers of g-C_3_N_4_ [35,39]. It can be seen that the characteristic g-C_3_N_4_ peak at 27° becomes broader and weaker with the increase in Pd and Rh content (Figure 1). For the sample 0.5% Pd/g-C_3_N_4_, a characteristic peak at 40.1° appears, which belongs to the (111) diffraction plane of metallic Pd (Figure 1b). For the rest of the rhodium- and palladium-containing samples, no metal peaks were found, probably due to low mass fraction of Rh and Pd.

The interlayer distance d_002_ for the all samples was ca. 3.22 Å, which is in good agreement with the data obtained for g-C_3_N_4_ [39]. The average crystallite size in the plane of the layer was 9.5–9.6 nm, and the average crystallite size in the direction perpendicular to the layer was 9.3–9.8 nm.

#### 3.1.2. UV–Vis Spectroscopy

**Figure 2:**
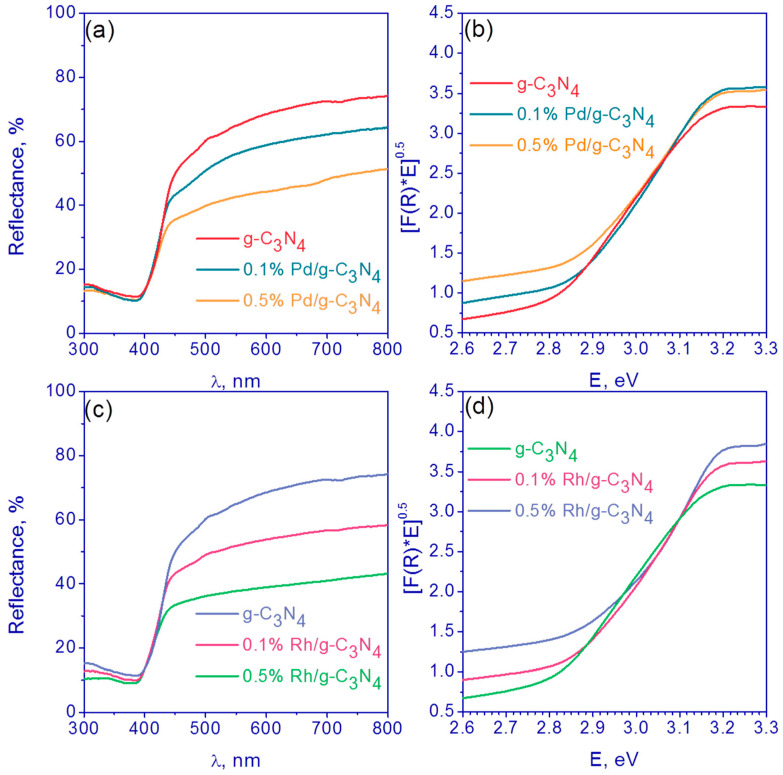
(**a**,**c**) Diffuse reflectance spectra and (**b**,**d**) Tauc plots of (**a**,**b**) pristine g-C_3_N_4_, Pd (0.1 and 0.5 wt.%)/g-C_3_N_4_ and (**c**,**d**) Rh (0.1 and 0.5 wt.%)/g-C_3_N_4_.

The optical properties of synthesized photocatalysts were investigated using UV–Vis spectroscopy (Figure 2). One can see that the absorption edge for all samples is ca. 400 nm (Figure 2a,c). Also, the deposition of noble metals promotes the adsorption of visible light in the range 400–800 nm. Figure 2b,d show the data transformation for pristine g-C_3_N_4_, Pd (0.1 and 0.5 wt.%)/g-C_3_N_4_ and Rh (0.1 and 0.5 wt.%)/g-C_3_N_4_ photocatalysts in Tauc’s coordinates for indirect semiconductors. It was shown that the deposition of metals has almost no effect on the band gap energy, which is in the range of 2.77–2.83 eV for all the samples.

#### 3.1.3. HR TEM Method

**Figure 3:**
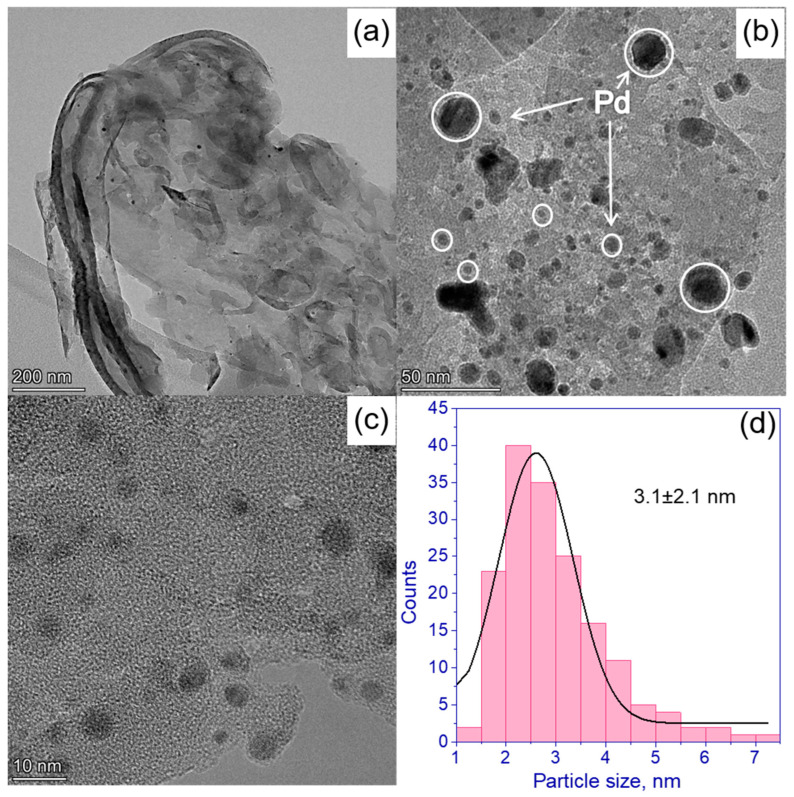
(**a**–**c**) HRTEM images of 0.5% Pd/g-C_3_N_4_ photocatalyst; (**d**) Pd particle size distribution calculated based on HRTEM data.

**Figure 4:**
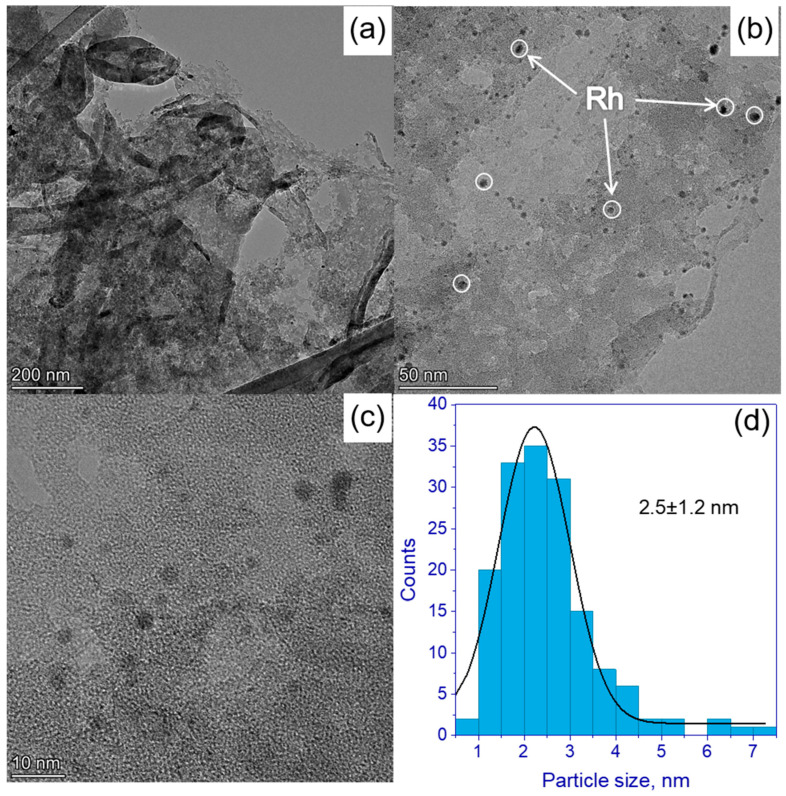
(**a**–**c**) HRTEM images of 0.5% Rh/g-C_3_N_4_; (**d**) Rh particle size distribution calculated based on HRTEM data.

High-resolution TEM images show the morphology of 0.5% Pd/g-C_3_N_4_ (Figure 3) and 0.5% Rh/g-C_3_N_4_ (Figure 4) photocatalysts and corresponding particle size distributions. Fourier transform of TEM images (Figure S1) reveals the interplanar distance of 3.3 Å, which is in good agreement with the interplanar distance determined from the XRD patterns (Figure 1) and with the previously published data [35,39,40]. One can see that both Pd and Rh particles have spherical shape. The average metal particles size was 3.1 ± 2.1 nm for Pd (Figure 3d) and 2.5 ± 1.2 nm for Rh (Figure 4d), respectively. The particle size distribution of both Pd and Rh includes particles ranging in size from 1 to 7 nm (Figure 3d and Figure 4d). In addition, it should be noted that palladium forms large clusters 20–30 nm in size, while rhodium is more evenly distributed over the surface of g-C_3_N_4_. We can assume that rhodium complexes are adsorbed on the g-C_3_N_4_ surface more uniformly, which is associated with the preferred structure of the complex Rh_2_(H_2_O)_8_(μ-OH)_2_](NO_3_)_4_∙4H_2_O compared to (Et_4_N)_2_[Pd(NO_3_)_4_.

HRTEM images were also used to confirm the presence of Pd and Rh in the synthesized photocatalysts (Figure S2). The observed distances for 0.5% Rh/g-C_3_N_4_ were equal to 2.19 and 1.91–1.92 Å, corresponding to Rh planes (111) and (200), respectively (PDF No. 5-685). A similar situation is observed for the 0.5% Pd/g-C_3_N_4_ photocatalyst. The interplane distances were equal to 2.28 and 2.09 Å, respectively, corresponding to the Pd planes (111) and (200) (PDF No. 46-1043).

#### 3.1.4. XPS Method

**Figure 5:**
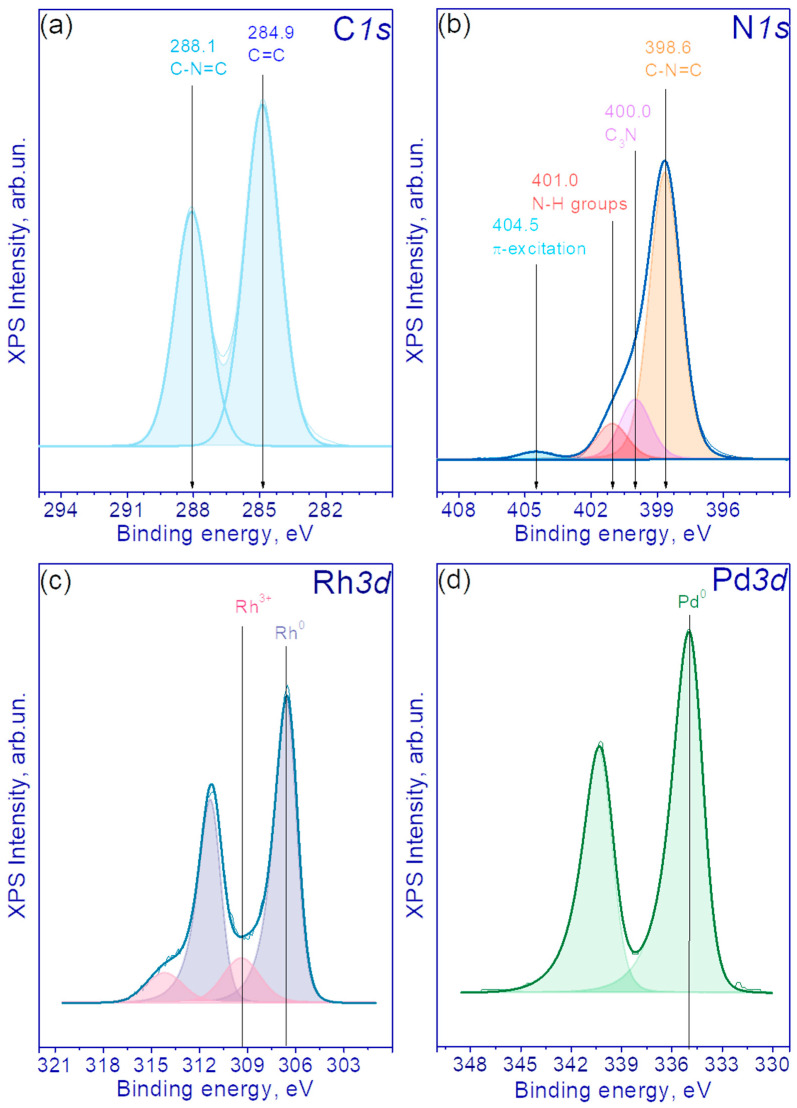
(**a**) The C1s and (**b**) N1s XPS spectra of pristine g-C_3_N_4_; (**c**) Rh3d spectra of 0.5%Rh/g-C_3_N_4_ and (**d**) Pd3d spectra of 0.5%Pd/g-C_3_N_4_.

The study of the chemical composition of the samples was carried out with the XPS technique. The XPS spectra of the catalysts revealed peaks corresponding to Pd, Rh, N, C and O. Figure 5a,b shows the C1s and N1s spectra of pristine g-C_3_N_4_. The C1s spectrum is well described by two peaks with binding energies of 284.9 and 288.1 eV. The first peak is characteristic of carbon-containing impurities present on the surface of the objects under study (often used to calibrate the binding energy scale). The second peak is characteristic of C1s g-C_3_N_4_ and corresponds to carbon forming bonds with nitrogen atoms in the g-C_3_N_4_ structure [41,42]. In the case of the N1s spectrum, four peaks are observed with binding energies of 398.6, 400.0, 401.0 and 404.5 eV. According to the literature data, the first peak refers to nitrogen atoms forming a C-N=C bond, the second one to N-(C)_3_ bonds with three carbon atoms, the third one to the N-H terminal groups and the fourth peak corresponds to an excited π bond [41].

Figure 5c shows the Rh3d spectra for the photocatalyst 0.5%Rh/g-C_3_N_4_. The Rh3d spectrum is described by one asymmetric Rh4f_7/2_–Rh4f_7/2_ doublet with a Rh3d_5/2_ binding energy of 306.5 eV and one symmetrical Rh4f_7/2_–Rh4f_7/2_ doublet with a Rh3d_5/2_ binding energy of 309.4 eV. In the literature, for metallic Rh, the binding energy of Rh4f_7/2_ is equal to 306.7–307.5 eV, and for Rh_2_O_3_ it lies in the range of 309.4–310.2 eV [28]. Thus, the doublet in the region of high binding energies refers to rhodium in the Rh^3+^ state, and the narrow asymmetric doublet in the lower binding energies region can be attributed to rhodium in the metallic state Rh^0^. According to the analysis, the proportion of Rh in the metallic state is 75%, and rhodium in the Rh^3+^ state is 25%, respectively. Figure 5d shows the Pd3d spectra of the 0.5% Pd/g-C_3_N_4_ photocatalyst. The Pd3d spectrum is well described by the Pd3d_5/2_-Pd3d_3/2_ doublet with Pd3d_5/2_ binding energy of 335.0 eV. For palladium in the metallic state, the Pd3d_5/2_ binding energy is 335.0–335.2 eV [32], and for PdO it is 336.6–337.7 eV [32,43]. Thus, in 0.5% Pd/g-C_3_N_4_ catalyst, Pd exists completely in the metallic state.

The atomic percentages of the surface for the samples 0.5% Rh/g-C_3_N_4_ and 0.5% Pd/g-C_3_N_4_ are shown in Table S2. It can be seen that the surface content of rhodium is much higher than in the case of palladium at the same weight content. The presented data on the surface content of rhodium and palladium once again confirm that in the case of the first metal, the deposition occurs with the formation of smaller particles with a good coverage of the g-C_3_N_4_ surface.

In general, from the characterization of photocatalysts, it can be concluded that 0.01–0.5% Pd/g-C_3_N_4_ and 0.01–0.5% Rh/g-C_3_N_4_ samples were obtained. In the case of the reduction of palladium from (Et_4_N)_2_[Pd(NO_3_)_4_] complex, it was possible to achieve a completely metallic state, whereas in the case of the use of [Rh_2_(H_2_O)_8_(μ-OH)_2_](NO_3_)_4_∙4H_2_O as a rhodium precursor, complete reduction of the metal was not achieved.

### 3.2. Photocatalyst Characterization

**Figure 6:**
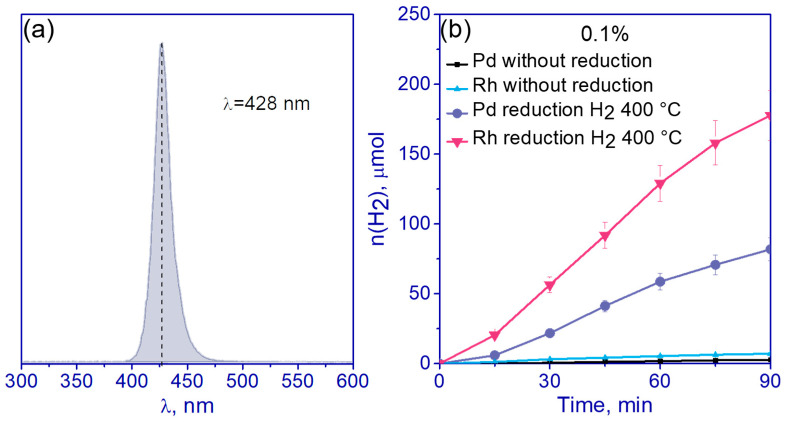
(**a**) Emission spectrum of 428 nm LED used as a light source; (**b**) the kinetic curves of photocatalytic hydrogen evolution over 0.1% Rh/g-C_3_N_4_ and 0.1% Pd/g-C_3_N_4_ without H_2_ reduction and with H_2_ reduction at 400 °C. Conditions: C(cat) = 0.5 g/L, 10 vol.% TEOA, C_0_(NaOH) = 0.1 M.

The activity of all synthesized samples 0.01–0.5% Pd/g-C_3_N_4_ and 0.01–0.5% Rh/g-C_3_N_4_, as well as the activity of pristine g-C_3_N_4_ and Pd- and Rh-containing samples before the reduction in hydrogen (with adsorbed complexes of palladium or rhodium after drying), was studied in the photocatalytic production of hydrogen in aqueous and aqueous alkaline solutions of TEOA. TEOA was chosen as a sacrificial agent as it helps to reduce photocorrosion of g-C_3_N_4_ because the amino groups of TEOA effectively bind to the surface of g-C_3_N_4_ [44,45]. Alkaline solution was used to facilitate proton abstraction during TEOA oxidation and to prevent charge recombination on the catalyst surface [46]. The photocatalytic activity was studied with the use of LED with a maximum emission at a wavelength of 428 nm as a light source (Figure 6a). Previously, it was shown that when using a diode with a maximum radiation in this region, the highest AQE was observed [35]. Note that over pristine g-C_3_N_4_, the rate was equal to zero both under neutral and alkali conditions. Figure 6b shows the kinetics of hydrogen formation for 0.1% Pd/g-C_3_N_4_ and 0.1% Rh/g-C_3_N_4_ samples without heat treatment and with H_2_ reduction at 400 °C.

One can see that the activity of photocatalysts with adsorbed complexes of rhodium and palladium is an order of magnitude lower than after the reduction of the corresponding complexes with H_2_ at 400 °C. As shown earlier for platinized photocatalysts, treatment with H_2_ at 400 °C is a necessary step for the formation of a metal cocatalyst for hydrogen evolution on the surface of g-C_3_N_4_ [35]. The next step in the study was to vary the mass fraction of metals from 0.01 to 0.5%. As was described before, [Rh_2_(H_2_O)_8_(μ-OH)_2_](NO_3_)_4_∙4H_2_O and (Et_4_N)_2_[Pd(NO_3_)_4_] were used as precursors of rhodium and palladium, respectively; reduction in H_2_ at 400 °C was used to obtain metallic particles for all samples. The data on activities of all the samples per 1 g of catalyst and per 1 g of noble metal are represented in Table 1.

**Figure 7:**
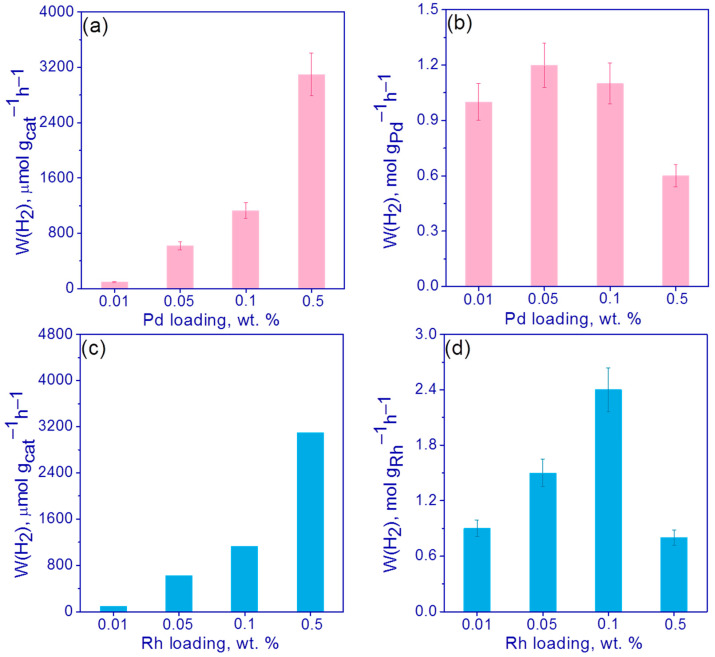
(**a**,**b**) Activity of 0.01–0.5% Pd/g-C_3_N_4_ and (**c**,**d**) 0.01–0.5% Rh/g-C_3_N_4_ photocatalysts in hydrogen evolution (**a**,**c**) per 1 g of catalyst and (**b**,**d**) per 1 g of metal. Conditions: C(cat) = 0.5 g/L, 10 vol.% TEOA, C_0_(NaOH) = 0.1 M, 428 nm LED.

The photocatalytic activities vs. metal loading are shown in Figure 7. As can be seen, the catalytic activity increases with the increase in a metal content for both Pd and Rh (Figure 7a,c), and the highest catalytic activities were observed for 0.5% Rh/g-C_3_N_4_ and 0.5%Pd/g-C_3_N_4_ photocatalysts (Figure 7a,c). The activity of 0.5% Rh/g-C_3_N_4_ photocatalysts was equal to ca. 3.9 mmol H_2_ per gram per hour and was about 25% higher than the activity of the photocatalyst 0.5% Pd/g-C_3_N_4_. Recalculation of activity per 1 g of metal showed that the maximum value and the most efficient use of the metal was observed for 0.1% Rh/g-C_3_N_4_ and was equal to 2.4 mol H_2_ per gram of Rh per hour (Figure 7d). This parameter was already twice that of the photocatalyst 0.1% Pd/g-C_3_N_4_.

Thus, it can be concluded that in the case of supported rhodium for the Rh/g-C_3_N_4_ photocatalysts, the use of Rh nanoparticles in the formation of hydrogen is more efficient than in the case of supported palladium for the photocatalysts Pd/g-C_3_N_4_. This may be due to the fact that the palladium deposited on the surface of the carbon nitride has a larger particle size compared to rhodium (Figure 3 and Figure 4). In addition, as can be seen from Figure 3b, palladium particles are collected into large aggregates, which can adversely affect the photocatalytic activity. The effect of metal nanoparticles—rhodium and palladium—as cocatalysts of hydrogen formation is likely similar. The deposition of metals leads to the separation of photogenerated charges due to the appearance of a Schottky barrier at the metal–semiconductor interface [8]. The main factor affecting the activity is most likely the formation of large agglomerates in the case of deposited rhodium. These large metal particles prevent the absorption of light by the semiconductor and can act as centers for the recombination of electron–hole pairs.

We compared numerical values obtained in this study with our previous results on platinized photocatalysts obtained with the (Bu_4_N)_2_[Pt(NO_3_)_6_] labile complex as a platinum precursor [35,45] as well as with previously published data on Pd/g-C_3_N_4_ [27] and Rh/g-C_3_N_4_ [26] photocatalysts. It has been shown that with similar methods of synthesis with the deposition of noble metals from labile precursor complexes, the highest activity in the series of Pt, Rh and Pd metals is observed for platinum. Thus, for the photocatalyst 0.5% Rh/g-C_3_N_4_ under study, the activity per gram of catalyst was 4 mmol g^−1^ h^−1^, and for the photocatalyst 0.5% Pt/g-C_3_N_4_ [35] it was 11 mmol g^−1^ h^−1^; the difference in the highest specific activity per gram of metal was also about 3 times higher for platinum photocatalysts (Table 1). Platinum is known as the most effective co-catalyst for the formation of hydrogen because Pt forms the highest Schottky barrier at the metal–semiconductor interface, due to the highest work function relative to other metals [8]. It is interesting to note that the activity of palladium photocatalysts Pd/g-C_3_N_4_ obtained using (Et_4_N)_2_[Pd(NO_3_)_4_] complex as a precursor of palladium is approximately on the same level as for photocatalysts for which ordinary palladium chloride acted as a precursor [27]. At the same time, Table 1 shows that the activity of rhodium photocatalysts Rh/g-C_3_N_4_ obtained using [Rh_2_(H_2_O)_8_(μ-OH)_2_](NO_3_)_4_∙4H_2_O complex as a precursor is orders of magnitude higher than the activity of photocatalysts Rh/g-C_3_N_4_ in which RhCl_3_ or Rh(acac)_3_ acted as a rhodium precursor [26]. In order to justify the choice of the precursor, the deposition of Pd and Rh (0.5 wt%) on the surface of g-C_3_N_4_ with the use of [Pd(NH_3_)_4_(NO_3_)_2_] and RhCl_3_ as precursors, respectively, was carried out. The activity in the photocatalytic hydrogen evolution was lower by ca. 2 and 6 times for 0.5% Pd/g-C_3_N_4_ and 0.5% Rh/g-C_3_N_4_ photocatalysts compared to similar samples synthesized with the use of (Et_4_N)_2_[Pd(NO_3_)_4_] and [Rh_2_(H_2_O)_8_(μ-OH)_2_](NO_3_)_4_∙4H_2_O precursors (Figure S3). Thus, the synthesis method proposed in this work for 0.01–0.5% Rh/g-C_3_N_4_ photocatalysts can be considered quite promising, especially considering the acceptable activity at low mass fractions of the metal.

**Figure 8:**
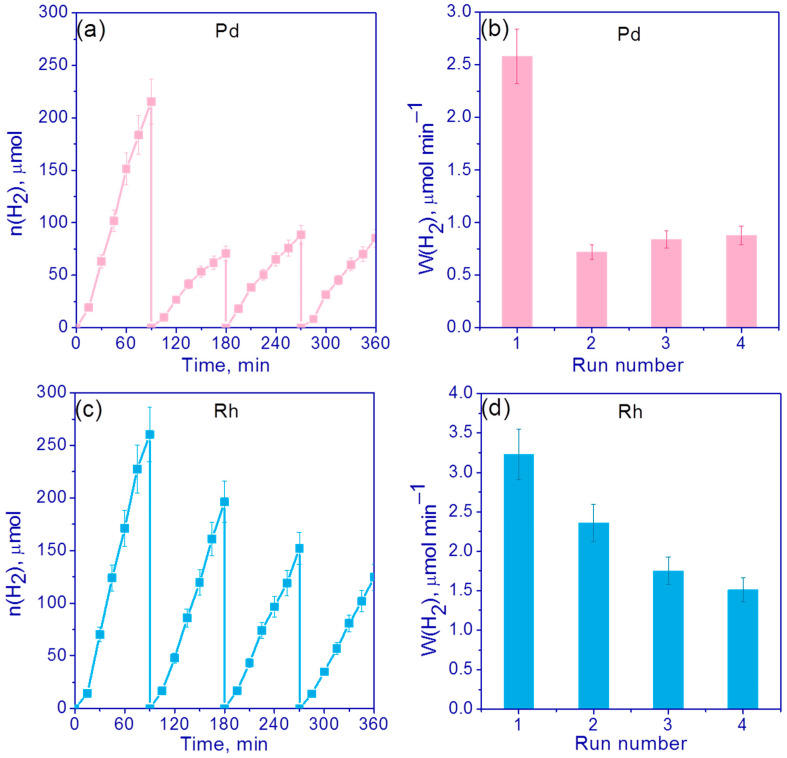
(**a**,**c**) The kinetic curves and (**b**,**d**) H_2_ evolution rates for photocatalysts (**a**,**b**) 0.5% Pd/g-C_3_N_4_ and (**c**,**d**) 0.5% Rh/g-C_3_N_4_. Conditions: C(cat) = 0.5 g/L, 10 vol.% TEOA, C_0_(NaOH) = 0.1 M, 428 nm LED.

Next, the stability of the most active photocatalysts, 0.5 wt.% Pd/g-C_3_N_4_ and 0.5 wt.% Rh/g-C_3_N_4_, was investigated in four runs in alkali (C_0_(NaOH) = 0.1 M) suspensions of TEOA, each of which lasted 1.5 h. After every 1.5 h of the reaction cycle, the suspension was purged with argon for 30 min. Figure 8a,c shows the kinetic curves and Figure 8b,d shows H_2_ evolution rates for the cyclic experiments.

**Figure 9:**
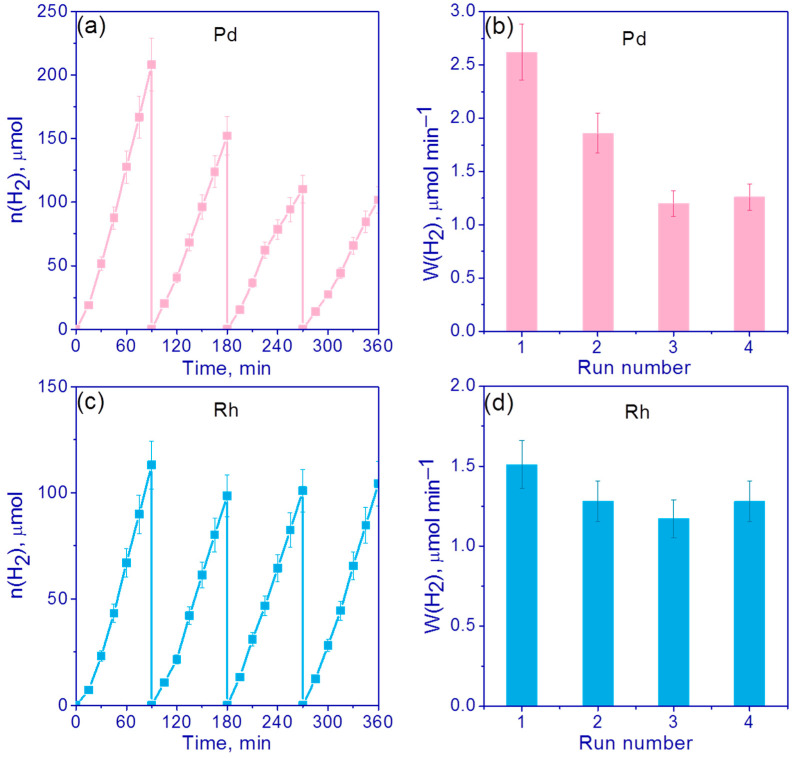
(**a**,**c**) The kinetic curves and (**b**,**d**) H_2_ evolution rates for (**a**,**b**) 0.5% Pd/g-C_3_N_4_ and (**c**,**d**) 0.5% Rh/g-C_3_N_4_. Conditions: C(cat) = 0.5 g/L, 10 vol.% TEOA, 428 nm LED.

In the case of a 0.5% Pd/g-C_3_N_4_ photocatalyst, the activity dropped sharply after the first run and then remained unchanged for three runs (Figure 8a,b). The drop in the activity was by approximately three times (Figure 8b). As can be seen, the deactivation of 0.5% Rh/g-C_3_N_4_ occurred gradually, and no sharp drop in activity is observed after the first cycle (Figure 8c,d). However, after the fourth cycle, the hydrogen evolution rate became 2 times lower compared to the first cycle (Figure 8d). Judging from the data of cyclic experiments, the decrease in activity in the case of photocatalysts with rhodium and palladium proceeded through different mechanisms. It can be assumed that the deactivation of the catalysts is most likely associated with the poisoning of active centers by TEOA oxidation products [35]. In order to reduce the influence of the resulting TEOA oxidation products, it was decided to study the stability of 0.5% Pd/g-C_3_N_4_ and 0.5% Rh/g-C_3_N_4_ under neutral conditions without addition of NaOH (Figure 9).

Figure 9c,d show that in the absence of NaOH, deactivation practically did not occur in the case of 0.5% Rh/g-C_3_N_4_ and amounted to only 15% (Figure 9c,d). However, in general, the activity was lower (Figure 9c,d) than in the experiments in alkaline media with addition of NaOH (Figure 8c,d). Thus, for the first run, the rate was 2 times lower compared to the experiments in the alkaline suspension of TEOA. In the case of 0.5% Pd/g-C_3_N_4_, the hydrogen evolution rate did not decrease compared to the experiments where NaOH was added into the reaction suspension; however, a twofold decrease in the rate after four runs was still observed (Figure 9a,b). We compared the surface content of elements before and after the photocatalytic tests for the samples 0.5% Pd/g-C_3_N_4_ and 0.5% Rh/g-C_3_N_4_ using the XPS technique (Table S2). It was shown that for 0.5% Rh/g-C_3_N_4_ photocatalysts, after the hydrogen evolution both in alkaline media and without NaOH addition, all rhodium became metallic with zero oxidation state; for both cases, the surface content of the metal fell by 2 times but remained rather high. In the case of tests in a 0.1 M NaOH medium, significant surface carbonization was observed, which is probably the cause of deactivation. For the 0.5 % Pd/g-C_3_N_4_ photocatalyst, we observed the decrease in surface Pd content only in alkaline media; however, the deactivation was observed both in the cases of alkaline and neutral media (Figure 8 and Figure 9).

In general, it should be noted that the deactivation mechanisms differ for the Rh/g-C_3_N_4_ and Pd/g-C_3_N_4_ photocatalysts, which is associated with different charge states of metals and their particle size distribution and requires further study.

## 4. Conclusions

In this research, a series of photocatalysts 0.01–0.5% Pd/g-C_3_N_4_ and 0.01–0.5% Rh/g-C_3_N_4_ was synthesized and characterized by a set of physicochemical methods. A distinctive feature of the synthesis was the use as precursors of such labile complexes as [Rh_2_(H_2_O)_8_(μ-OH)_2_](NO_3_)_4_∙4H_2_O and (Et_4_N)_2_[Pd(NO_3_)_4_] with further reduction in H_2_ at 400° in order to obtain metallic particles of rhodium and palladium, respectively. It has been shown that in the case of the reduction of palladium from (Et_4_N)_2_[Pd(NO_3_)_4_] complex, it is possible to achieve a completely metallic state, whereas in the case of the use of [Rh_2_(H_2_O)_8_(μ-OH)_2_](NO_3_)_4_∙4H_2_O, rhodium exists in the Rh^0^ and Rh^3+^ states.

It was shown that the activity in the hydrogen evolution from aqueous solutions of TEOA under visible light for the 0.01–0.5% Rh/g-C_3_N_4_ series is higher than in the case of the 0.01–0.5% Pd/g-C_3_N_4_ photocatalysts. For the Rh/g-C_3_N_4_ photocatalysts, the use of Rh nanoparticles in the formation of hydrogen is likely more efficient than in the case of supported palladium for the photocatalysts Pd/g-C_3_N_4_ because palladium particles are collected into large aggregates, which can adversely affect the photocatalytic activity. The highest activity per gram of photocatalysts was possessed by the sample 0.5% Rh/g-C_3_N_4_ and was equal to ca. 3.9 mmol H_2_ g^−1^ h^−1^; the highest activity per gram of metal was possessed by 0.1% Rh/g-C_3_N_4_ and was equal to 2.4 mol H_2_ g_Rh_^−1^ h^−1^. It is interesting to note that the activity of the Rh/g-C_3_N_4_ photocatalysts obtained using [Rh_2_(H_2_O)_8_(μ-OH)_2_](NO_3_)_4_∙4H_2_O complex as a precursor is orders of magnitude higher than the activity of photocatalysts Rh/g-C_3_N_4_ in which RhCl_3_ or Rh(acac)_3_ acted as a rhodium precursor [20]. Thus, the synthesis method proposed in this work for 0.01–0.5% Rh/g-C_3_N_4_ photocatalysts can be considered quite promising, especially considering the acceptable activity at low mass fractions of the metal.

## Figures and Tables

**Table 1:** Photocatalytic properties of the synthesized samples and comparison with the literature data.

No.	Photocatalyst	Metal Weight Content, %	Catalytic Activity, µmol g_cat_^−1^ h^−1^	Catalytic Activity, mol g_Metal_^−1^ h^−1^	AQY, %	Ref.
1	g-C_3_N_4_	-	<1	0	0	
Pd/g-C_3_N_4_						
2	Pd/g-C_3_N_4_	0.01	96	1.0	<0.1	This study
3		0.05	620	1.2	0.4	
4		0.1	1080	1.1	0.7	
5		0.5	3100	0.6	1.8	
6	Pd/g-C_3_N_4_ (from [Pd(NH_3_)_4_(NO_3_)_2_])	0.5	1730	0.3	1.0	
7	Pd/g-C_3_N_4_ (from PdCl_2_)	0.5	3300	0.7	-	
8		0.5	7600	1.5	2.4 (400 nm)	[27] *
Rh/g-C_3_N_4_						
9	Rh/g-C_3_N_4_	0.01	88	0.9	<0.1	This study
10		0.05	732	1.5	0.4	
11		0.1	2400	2.4	1.4	
12		0.5	3900	0.8	2.3	
13	Rh/g-C_3_N_4_ (from RhCl_3_)	0.5	610	0.1	0.4	
14	Rh/g-C_3_N_4_ (from RhCl_3_)	0.25	14.9	0.006	-	[26] **
15		0.23	13.3	0.006		
16	Rh/g-C_3_N_4_ (from Rh(acac)_3_	0.34	10.7	0.003		
17		0.27	3.8	0.001		
Pt/g-C_3_N_4_						
18	Pt_0_._1_/g-C_3_N_4_	0.1	8520	8.5	5.0	[35] ***
19	Pt_0_._5_/g-C_3_N_4_	0.5	11,300	2.2	6.6	
20	Pt_0_._1_/g-C_3_N_4_	0.1	7560	7.6	4.4	[47] ***
21	Pt_0_._5_/g-C_3_N_4_	0.5	8520	1.7	5.0	

* Substrate—10 vol.% TEOA, λ = 400 nm. ** Substrate—10 vol.% methanol, Xe arc lamp with cut-off filter λ = 420 nm. *** Conditions are the same as for photocatalysts No. 1–6 and 7–10.

## Data Availability

The data presented in this study are available on request from the corresponding author.

## Supplementary Materials

The following supporting information can be downloaded at: https://www.mdpi.com/article/10.3390/nano13152176/s1, Figure S1. (a,b) HRTEM images of 0.5% Rh/g-C_3_N_4_ and Fourier transform from the highlighted image. Figure S2. (a,b) HRTEM images of 0.5% Rh/g-C_3_N_4_ and 0.5% Pd/g-C_3_N_4_. Figure S3. H_2_ evolution rates for photocatalysts 0.5% Pd/g-C_3_N_4_ and 0.5% Rh/g-C_3_N_4_ synthesized from different metal precursors. Table S1. Actual metal content in the samples determined with ICP AES. Table S2. Surface element atomic concentration calculated based on XPS data of fresh 0.5% Pd/g-C_3_N_4_ and 0.5% Rh/g-C_3_N_4_ photocatalysts and these photocatalysts after cyclic runs in 0.1 M NaOH and water (Figure 8 and Figure 9).
