# Supplementary material for: Photocatalysts Based on Graphite-like Carbon Nitride with a Low Content of Rhodium and Palladium for Hydrogen Production under Visible Light
Source: Nanomaterials (Basel). 2023 Jul 26;13(15):2176. doi: 10.3390/nano13152176 (PMC10421291; doi:10.3390/nano13152176)
Supplement: Supplementary file 1 [file nanomaterials-13-02176-s001.zip › nanomaterials-2510619-supplementary.pdf]

Supplementary materials for

Photocatalysts based on graphite-like carbon nitride with a low content of rhodium and palladium for the hydrogen production under the visible light

Angelina V. Zhurenok [1], Danila B. Vasichenko [1,2], Semen N. Berdyugin [2], Evgeny Yu. Gerasimov [1], Andrey A. Saraev [1], Svetlana V. Cherepanova [1], Ekaterina A. Kozlova [1,*]

[1] Federal Research Center, Boreskov Institute of Catalysis SB RAS, Lavrentieva Ave. 5, Novosibirsk 630090, Russia; angelinazhurenok@gmail.com (A.V.Z.); vasilchenko@niic.nsc.ru (D.B.V.); gerasimov@catalysis.ru (E.Y.G.); asaraev@catalysis.ru (A.A.S.); svch@catalysis.ru (S.V.C.)
[2] Nikolaev Institute of Inorganic Chemistry, Siberian Branch of the Russian Academy of Science, Novosibirsk 630090, Russia; berdyugin@niic.nsc.ru
* Correspondence: kozlova@catalysis.ru

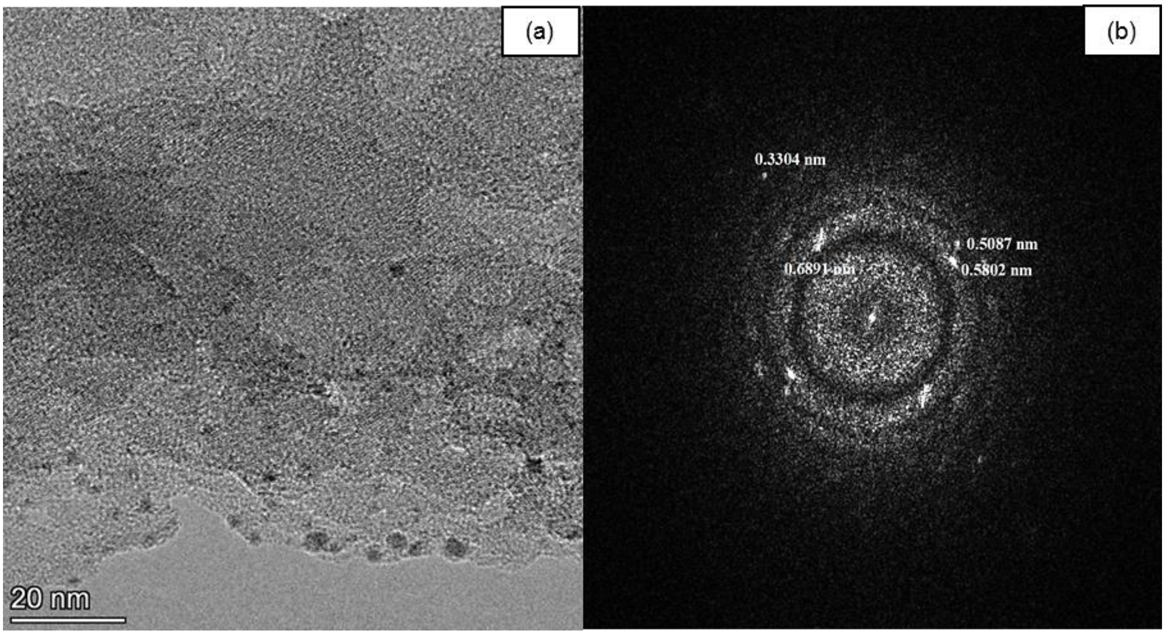

Figure S1. (**a**,**b**) HRTEM images of 0.5% Rh/g-C_3N_4 and Fourier transform from the highlighted image

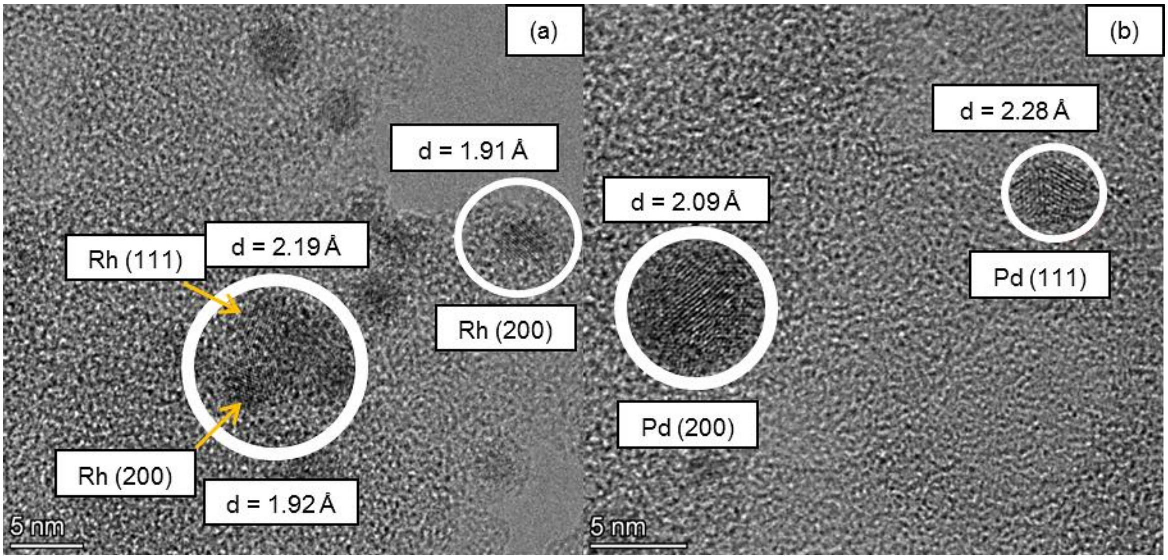

Figure S2. (**a**,**b**) HRTEM images of 0.5% Rh/g-C_3N_4 and 0.5% Pd/g-C_3N_4

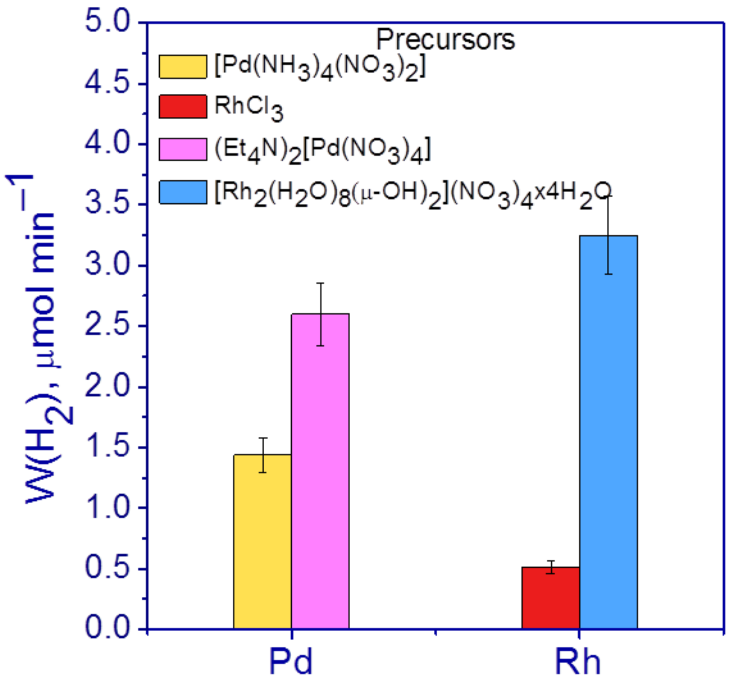

Figure S3. H_2 evolution rates for photocatalysts 0.5% Pd/g-C_3N_4 and 0.5% Rh/g-C_3N_4 synthesized from different metal precursors.

Table S1. Actual metal content in the samples determined with ICP AES

**Sample**	**Pd content, wt.%**	**Rh content, wt.%**
0.1% Pd/g-C_3N_4	0.093 ± 0.009	-
0.5% Pd/g-C_3N_4	0.45 ± 0.05	-
0.5% Pd/g-C_3N_4 ([Pd(NH_3)$_4$(NO_3)$_2$])	0.053 ± 0.05	-
0.1% Rh/g-C_3N_4	-	0.13 ± 0.01

Table S2. Surface element atomic concentration calculated based on XPS data of fresh 0.5% Pd/g-C_3N_4 and 0.5% Rh/g-C_3N_4 photocatalysts and these photocatalysts after cyclic runs in 0.1 M NaOH and water (Figures 8-9).

**Catalyst**	%, C (C_3N_4)	%, C (carbon)	%, N (C_3N_4)	%, O	%, Pd
0.5%Pd-fresh	27.4	32.3	34.3	6.0	0.02
0.5%Pd-NaOH	27.0	33.6	31.8	7.6	0.01
0.5%Pd-water	25.6	38.1	27.4	8.9	0.02
	%, C (C_3N_4)	%, C (carbon)	%, N (C_3N_4)	%, O	%, Rh
0.5%Rh-fresh	24.0	40.7	26.5	8.6	0.22
0.5%Rh-NaOH	20.9	52.6	15.0	11.4	0.10
0.5%Rh-water	25.6	39.4	26.4	8.5	0.09
